# Dopamine, Prediction Error and Beyond

**DOI:** 10.1177/1073858420907591

**Published:** 2020-04-26

**Authors:** Kelly M. J. Diederen, Paul C. Fletcher

**Affiliations:** 1 Department of Psychosis Studies, Institute of Psychiatry, Psychology and Neuroscience, King’s College London, London, UK; 2 Department of Psychiatry, University of Cambridge, Cambridge, UK; 3 Cambridgeshire and Peterborough NHS Foundation Trust, Cambridge, UK; 4 Wellcome Trust MRC Institute of Metabolic Science, University of Cambridge, Cambridge Biomedical Campus, Cambridge, UK

**Keywords:** dopamine, prediction errors, brain, psychiatry, learning

## Abstract

A large body of work has linked dopaminergic signaling to learning and reward processing. It stresses the role of dopamine in reward prediction error signaling, a key neural signal that allows us to learn from past experiences, and that facilitates optimal choice behavior. Latterly, it has become clear that dopamine does not merely code prediction error size but also signals the difference between the expected value of rewards, and the value of rewards actually received, which is obtained through the integration of reward attributes such as the type, amount, probability and delay. More recent work has posited a role of dopamine in learning beyond rewards. These theories suggest that dopamine codes absolute or unsigned prediction errors, playing a key role in how the brain models associative regularities within its environment, while incorporating critical information about the reliability of those regularities. Work is emerging supporting this perspective and, it has inspired theoretical models of how certain forms of mental pathology may emerge in relation to dopamine function. Such pathology is frequently related to disturbed inferences leading to altered internal models of the environment. Thus, it is critical to understand the role of dopamine in error-related learning and inference.

## Introduction

Dopamine is a critical modulatory neurotransmitter. Acting within distinct pathways, it is involved in a wide range of functions, including the control of movement, motivation, reward processing, and learning. Its perturbation has been linked to profound neurodegenerative and psychiatric impairments (Bissonette and Roesch 2016). In the past, dopamine’s association with “happiness” or “pleasure” has been emphasized in view of its role in the prediction, anticipation, and approach behavior toward rewarding outcomes (Arias-Carrión and Pŏppel 2007). Indeed, there is overwhelming evidence that dopamine guides learning about reward outcomes, by keeping track of violations in our expectations, called prediction errors (PEs) (Schultz 2016a). However, dopamine may have a role in the signaling of PEs that are not directly related to reward (Friston 2010). Here, we provide an overview of the evidence relating dopaminergic function to reward learning and discuss emerging work that suggests a crucial role for dopamine in predicting any future outcomes. In doing so, we consider how it may be a key contributor to setting up a model of associative regularities in the environment as a basis for flexible inference and how disruption in this role may parsimoniously explain key symptoms of neuropsychiatric disorders (Fletcher and Frith 2009; Sterzer and others 2018).

### Prediction Errors

Predicting the outcomes of our actions is crucial for effective decision making and behavior. An efficient mechanism for learning is to keep track of violations in our expectations, termed PEs (outcome expected – outcome received) (Schultz 2016a). These errors effectively allow us to predict which outcomes are likely to be available at a particular time, and to guide our choices toward optimal behaviors. PEs are underpinned by dedicated neural signals which drive learning about outcomes in the domains of perception, motor function, punishment and reward (Den Ouden and others 2012). Reward PEs (RPEs) differ from sensory and motor PEs in that as well as engendering surprise (referred to as *unsigned* PEs), they indicate whether outcomes were better or worse than expected, resulting in positively and negatively *signed* PEs (Den Ouden and others 2012). Signed and unsigned PEs are (at least partially) underpinned by separate neural substrates (Fiorillo 2013). While the neurotransmitter dopamine has consistently been shown to play a major in the encoding of RPEs (Schultz 2016a), its role in other domains is less clear.

#### Rewards

Neuroscience research broadly defines rewards as any positive, or pleasurable, outcomes, that we are motivated to obtain and that we will work for (Schultz 2016a). To determine the sign (positive/negative), and size of RPEs we need to know how much individuals value specific rewards (Levy and Glimcher 2012). Primary rewards, including food, drink, and sex, are innately valuable due to their intrinsic survival properties. By contrast, indirect rewards such as money, derive their positive value from their association (conditioned reinforcement) with pleasurable outcomes (Wise 2002). Primary and secondary rewards are associated with similar behaviors (e.g., choices) and dopamine responses, compatible with the idea that the brain transforms all rewards onto a single scale of value that facilitates decision making when different actions may procure different types of rewards (Lak and others 2014).

#### The Dopaminergic System

**Figure 1.**
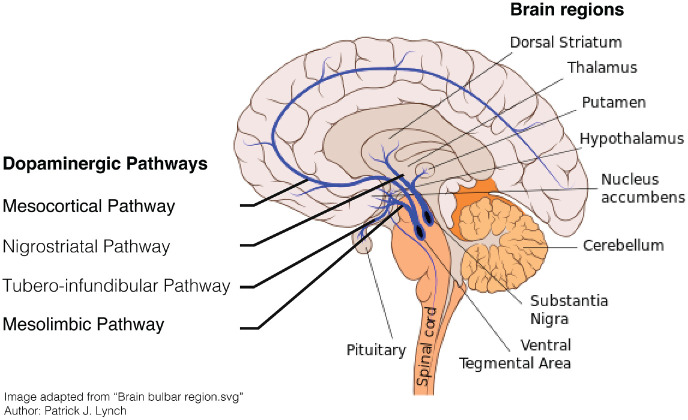
The dopaminergic system in the human brain.

Dopamine is synthesized by dopamine neurons and thence transported via axonal projections widely throughout the brain. Although dopamine has multiple functions, the brain contains relatively few dopamine neurons; ~400,000 in the human brain which accounts for ~1% of the total neuronal population (Arias-Carrión and Pŏppel 2007). The majority of dopamine neurons are located in two small nuclei in the midbrain called the ventral tegmental area (VTA), and the substantia nigra (SN), which has two subnuclei called the pars compacta (SNc) and pars reticulata (SNr; Nair-Roberts and others 2008). The latter two so-called because of the presence of the dark pigment melanin within the dopamine neurons (Halliday and Törk 1986). Four functionally distinct dopamine projections can be distinguished (Fig. 1). RPE signaling is primarily facilitated by the mesolimbic pathway, which transmits dopamine from the VTA to the nucleus accumbens (NA) in the ventral striatum. By contrast, the nigrostriatal pathway, which connects the SNc to the dorsolateral striatum, and the premotor/motor cortex is thought to facilitate action-selection of the most rewarding action (García-García and others 2017).

#### Measuring Dopaminergic Function

In animals, the phasic (fast spiking) and tonic (slower responses) of VTA/SN dopamine neurons can be measured directly using single cell electrophysiology (Schultz and others 1997). These phasic dopamine responses lead to dopamine release in the NA, which can be measured at a lower temporal resolution using fast-scan cyclic voltammetry and microdialysis (Clark and others 2009; Hart and others 2014). Indirect measurements in animals consist of electrical stimulation of dopamine neurons and administration of drugs that act on the dopaminergic system (Olds and Milner 1954). More recently, optogenetic stimulation has been used in rodents and monkeys to directly stimulate dopamine neurons (Kim and others 2012; Stauffer and others 2016).

**Figure 2.**
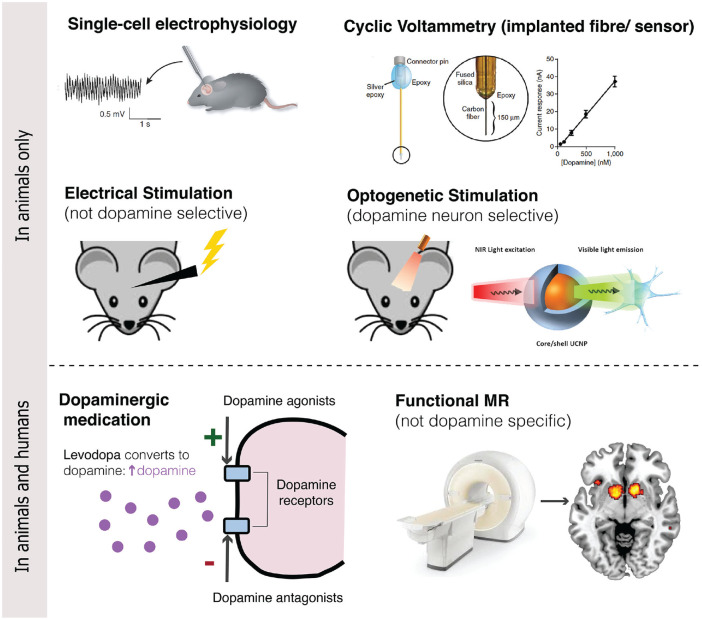
Techniques for investigating dopamine and prediction errors.

In humans, brain responses in the VTA/SN are mainly investigated using functional neuroimaging techniques (sometimes in conjunction with pharmacological challenges) such as functional MRI (fMRI) and position emission tomography (PET; Düzel and others 2015). fMRI measures changes in blood oxygen level, a proxy for neural activity, on a timescale of seconds. The low temporal and spatial resolution, however, make it difficult to determine whether observed signals reflect dopaminergic signaling as the VTA/SN are only partly made up of dopamine neurons, and the exact location of these nuclei varies across individuals (Düzel and others 2015). Although PET allows for non-invasive measurement of dopaminergic activity in humans, its temporal resolution is insufficient to draw direct comparisons to animal electrophysiological studies (Heiss 2009). In this review, we will discuss and integrate findings obtained using each of these techniques (Fig. 2), while considering the challenges in doing so.

#### RPEs and Reinforcement Learning

The idea of RPEs has long been central to ideas of classical and instrumental conditioning and to reinforcement learning (RL) generally. RL has its roots in the seminal work of Ivan Pavlov on classical (Pavlovian) conditioning in dogs (Pavlov and Anrep 1982), as well as in machine learning. Pavlov used the term reinforcement to describe the strengthening of the association between a reward (unconditioned stimulus [US]) and the conditioned stimulus (CS; e.g., sounding a bell). Repeated pairings of the CS with the US allowed Pavlov’s dogs to learn to predict the availability of a reward (food) when they heard a bell, as indicated by the CS—salivation on hearing the bell.

Whereas Pavlov focused on situations in which the outcome (food) followed the conditioned stimulus (bell) irrespective of any behavioral reaction, Edward Thorndike (Thorndike 1898) and Burrhus Frederic Skinner (Skinner 1938) studied what has come to be known as instrumental or operant conditioning, in which the animal’s behavior determines whether the unconditioned stimulus is presented (Skinner 1963). A now famous experiment included placing hungry cats in an enclosed container, which Skinner referred to as a puzzle box, from which they had to escape in order to reach food. The first time a cat was placed in this situation it escaped only after it made the right action (pressing a lever) by chance. The time it took to perform this action decreased each time it was returned to the box, suggesting that the cat was learning, or, specifically, that the useful action was being reinforced. While classical (or Pavlovian) and instrumental conditioning entail rather different experimental set-ups, they are tightly related. Stimuli associated with reward through classical conditioning come to motivate behavior generally, and can, more specifically, motivate particular behaviors that have been learned to be associated with the reward that they predict a phenomenon referred to as Pavlovian to Instrumental transfer (de Wit and Dickinson 2009; Estes 1948).

With regard to the involvement of RPE in RL, one key observation was that, if the PE is absent, learning does not occur even when a cue is strongly associated with an outcome. This is famously demonstrated in Kamin’s blocking effect (Kamin 1969), in which a previously learned cue-outcome association (A → X) blocks the acquisition of learning when a new cue is added (AB → X). In this case, there is no PE to AB-X because A already predicts X and so, though B is associated with X, the association is not reinforced. This remarkable observation underpins formal RL models (see Box 1).

**Box 1.**

**Reinforcement Leaning (RL).**

**RL.** Formal RL models foster a more mechanistic understanding of the different computations that a neural system must solve to translate to changes in behavior. A first formal (computational) model of RL was developed by Robert Rescorla and Allan Wagner, termed the Rescorla-Wagner (RW) model (Rescorla and Wagner 1972), which specifies that learning slows as the reinforcer (reward) becomes more predicted as a function of decreasing RPEs:

(1) $$y_{n}=y_{n-1}+\alpha *\delta_{n}$$

Here, predictions of reward value (y) are updated iteratively (n) as a function of the size of the RPE (δ) and a constant, termed the learning rate (α) that determines the weight attributed to PEs to drive learning. if we expect to get £10, but receive £16 instead, we will have a PE of £6. If we have a learning rate of 0.5, we will update our next prediction of reward to equal £13 (£10 + 0.5 * 6). When the PE equals zero no more learning occurs (Niv and Schoenbaum 2008; Schultz and Dickinson 2000).

The RW model lacks a consideration of time, assuming that predictions of reward are specific to each individual trial and that trials are discrete. Noting this, Sutton and Barto (1998) introduced the idea that predictions are based on all future expected rewards (within a particular environment), with the additional feature that temporally closer rewards have more value than those in the more distant future. This led to the so-called temporal difference model (Sutton and Barto 1998), which is a form of dynamic programming, to include a discount factor (γ) in their calculation of the PE which determines the extent to which rewards that arrive earlier are more important than rewards that arrive later on:

(2) $$\delta_{n}=r_{n}+\gamma {\hat{V}}_{n+1}\;1-\;{\hat{V}}_{n}$$

where the PE (δ) on a specific trial indicate the difference between the expected value of all future reward ($\hat{V}$).

Another strategy for weighting earlier outcomes, and hence PEs, more than later ones, is to reduce the learning rate as trials progress. Decreasing your rate of learning as time progress is sensible as one’s predictions become more reliable (and hence new outcomes less informative) as time progresses. Pearce and Hall (1980) allowed the learning rate to decrease across trials ($\alpha_{n}$) as a function of the absolute PE (|δ|) - which signals the extent to which previous predictions were wrong - and the learning rate on the previous trial, and an individually determined discount factor (*γ*):

(3) $$\alpha_{n}=\gamma \left|\delta_{n-1}\right|+\left(1-\gamma \right)\alpha_{n-1}$$

where the recursive process is initialized with the initial learning rate $\alpha_{0}=\alpha$.

**Extending RL Models to Decision Making.** The above models speak directly to the relationship between cues and (predicted) outcomes. Models based on similar principles have extended the ideas to instrumental conditioning, where the context-dependent value of different action options must be tracked and used to optimize choice behavior. One simple but powerful instance of this is the Q-learning model (Dickinson and Balleine 1994; Sutton and Barto 1998). This is closely related to RL models based on classical condition as the relationship between choices and reward values is learned via the PE (δ). For each pair of stimuli, *A* and *B*, the model estimates the expected values of choosing *A*(*Qa*) and choosing *B*(*Qb*), on the basis of individual sequences of choices and outcomes. This value, termed a *Q* value, is essentially the expected reward obtained by taking that particular action. After every trial *n* > 0 the value of the chosen stimulus is updated according to the following rule:

(4) $$QA_{n+1}=QA_{n}+\alpha *\;\delta_{n}$$

and the PE is calculated using the following formula to indicate the difference between the predicted value of the chosen option and the maximum discounted future *Q* value:

(5) $$\delta_{n}=r_{n}-Q_{n}$$

Given the *Q* values, the associated probability of selecting each action is then estimated by implementing a softmax rule (see Sutton and Barto, 1998 for examples). The softmax rule has two parameters one denoting the learning rate and the second denoting the (inverse) temperature. The temperature specifies the noise or randomness in choice behavior.Importantly, in all these models, learning only occurs if PEs are valenced, and they thus do not allow for learning associations of complex associative structures in the presence of non-RPEs.

In addition to the “model-free” RL models described above, there is a separate set of more flexible “model-based” RL models, which state that individuals build a cognitive model of environmental contingencies to allow for forward planning to identify the most rewarding options (Dickinson and Balleine 2002). Here, individuals evaluate possible actions by searching a cognitive model that represents the current *state* of the environment (e.g., the door is open), the likelihood that a reward will occur in this state, and how a decision may change the state (e.g., the door will close). Optimal decision making therefore requires individuals to predict future states, which can be learned from state PEs (Gläscher and others 2010).

#### Determining the Value of Rewards

Ultimately, the (model-free) RL models relate RPEs and learning to the value we assign to a particular reward (Sutton and Barto 1998). The question of how and why value is assigned is enormously complex. To identify the value of an expected reward individuals must integrate information on different reward attributes including its type, magnitude, probability, and timing (Lak and others 2014; Padoa-Schioppa 2011). Whereas reward value typically increases as the magnitude, probability, and temporal proximity increase, the weighting of each of these reward attributes varies across time and individuals. For instance, hunger increases the value of even a small or bland food reward. In addition, reward preferences vary across individuals, and depend on personality characteristics such as attention, motivation, patience and willingness to take a gamble (Padoa-Schioppa 2011). In general, people are risk avoidant, that is, they prefer smaller “safe” rewards over a gamble that can result in a larger, risky reward, but preference varies both across individuals and conditions (e.g., we are more likely to take a gamble when the amount of money at stake is small). Similarly, monkeys become more risk aversive (for juice rewards) when they are thirsty (Yamada and others 2013). In addition, our preference also depends on our ability to accurately learn about each of these reward characteristics and it has been shown that subjects’ estimate of probabilities tends to be distorted (Stauffer and others 2015; Tobler and others 2008; Tversky and Kahneman 1974).

These insights have led researchers to investigate rewards in terms of subjective rather than objective values (Bartra and others 2013; Kahneman and Tversky 2013). Subjective values can be determined by an individual’s choice behavior when asked to make a set of iterative choices between different options to determine the relative value of different rewards (Luce 2012; Taylor and Creelman 1967). The probability of choosing one option over others denotes the predicted value that subjects have attached to the available options. Crucially, choices are based on predictions of outcomes, which can be obtained through learning as the result of a choice is frequently not explicitly available to individuals (Cartoni and others 2013). In humans, we can additionally ask how much they prefer each reward or let them “play” in so-called first and second prize auctions in which people indicate how much of an endowment they are willing to pay for a particular reward (Becker and others 1964).

#### Dopamine and RPE Coding

In 1997, a clear neuronal substrate of RPEs was observed (Schultz and others 1997). Schultz and colleagues showed that dopamine neurons in the midbrain VTA/SN changed their firing rate in response to rewards, and to (conditioned) cues that are predictive of rewards. Specifically, dopamine neurons in the VTA/SN of macaque monkeys increased their firing rates for unexpected, but not expected, juice rewards. When a reward was preceded by a predictive visual stimulus, firing occurred in response to the stimulus but not the reward. This can be explained in terms of the predictive stimulus signaling a positive RPE and suggests that the visual cue had come to acquire properties of the reward itself. Moreover, when expected rewards were omitted the dopamine neurons showed a reduction in firing rates (indicating a negative PE). Overall, the findings clearly demonstrated that dopaminergic neurons did not respond to receiving a reward per se, but rather that these neurons tracked the violation in expected rewards. The authors furthermore showed that the observed dopamine responses obeyed the rules of RL models which provided further evidence for their key role in error-dependent learning. Numerous studies since then, including the measurement of dopamine release in the NA in animals, have proved compatible with these observations (Bayer and Glimcher 2005; Hart and others 2014). Importantly, it has recently been established that dopamine neurons do not inherit the RPEs from upstream regions but are directly involved in the computation of these PEs (Watabe-Uchida and others 2017).

In humans, the hypothesized role of dopamine in RPE signaling has been strongly supported by studies using neuroimaging and pharmacological dopaminergic manipulations. Using high-resolution fMRI, D’Ardenne and others (2008) studied the small VTA/SN nuclei in the human midbrain, as well as the NA. Activation occurred in response to unexpectedly large or small monetary rewards but not to rewards that were fully expected. Although these results do not necessitate that the observed PE signals were dopamine-dependent, complementary studies have shown that human PE signaling is modulated by administering (single) doses of dopaminergic agents to healthy individuals. Pessiglione and others (2006) found that the magnitude of RPE signals increased in the ventral striatum/NA of individuals who received levodopa (L-DOPA; a metabolic precursor of dopamine thought to increase dopamine signaling), compared to individuals who received haloperidol (a dopamine antagonist). Participants who received L-DOPA also won more money on the task, as they had learnt to choose the most rewarding option more frequently, suggesting that the elevated dopamine-dependent RPE drove improvements in learning, which optimized participants’ decisions.

**Figure 3.**
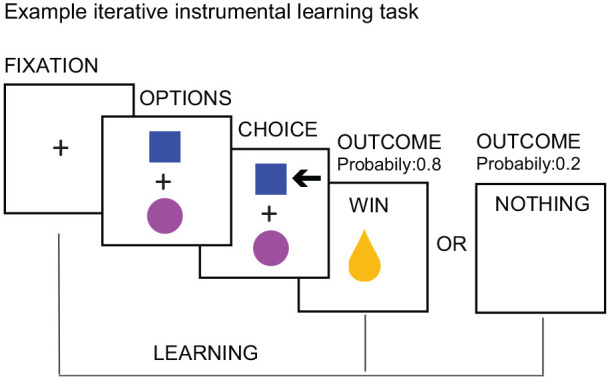
Example experiment for investigating reward prediction errors.

**Figure 4.**
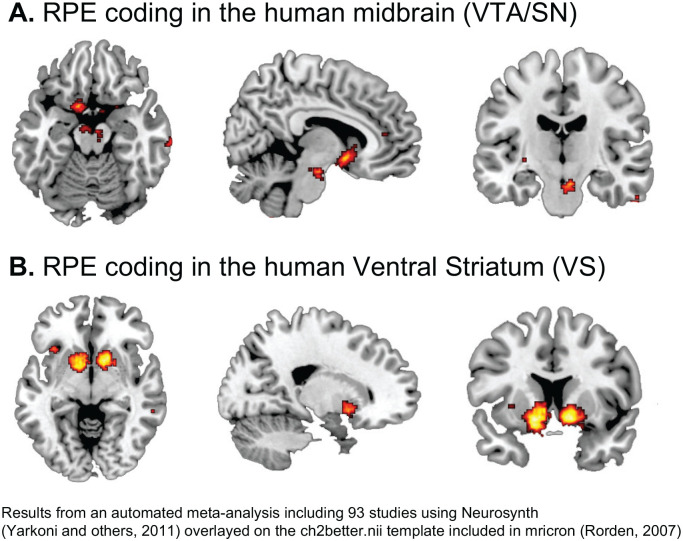
Reward prediction error (RPE) coding in the human brain.

**Figure 5.**
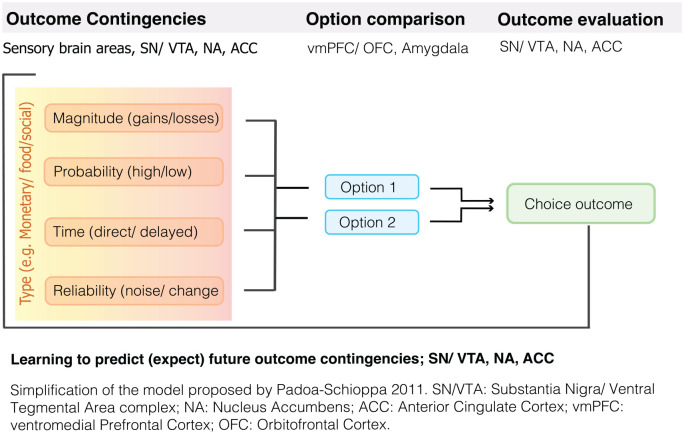
Reward learning and decision making.

A large number of studies have confirmed the role of the VTA/SN and the striatum in the signaling of RPEs across both direct and indirect rewards (Garrison and others 2013; Sescousse and others 2013). See Figure 3 for an example of an RPE experiment in humans. Importantly, in humans the striatum was the key brain area encoding PEs (Fig. 4) in both instrumental and classical conditioning/reinforcement (Garrison and others 2013). Studies in humans and animals alike have further established that the dopaminergic system integrates information about different reward characteristic, including the expected type (e.g., food or money), magnitude, probability, and time of reward (Fig. 5) to calculate RPEs (Diederen and others 2016; Lak and others 2014; O’Doherty and others 2003; Tobler and others 2005).

In addition, recent work in mice revealed that dopamine RPEs are sensitive to beliefs about the (model-based) “state” that an animal is in (Starkweather and others 2017). Each trial consisted of (1) a cue-reward state where the time until reward was drawn from a Gaussian distribution and (2) an interstimulus interval (ISI) state. In one task, odor cues predicted reward in 90% of trials, which meant that the transition from the cue-reward state to the ISI state was unobservable or *hidden*, and that longer cue-reward delays increased the belief that reward was omitted and that a state transition had occurred. Optogenetically identified dopamine neurons showed the highest responses to the latest rewards, suggesting that animals had inferred a state transition (to the ISI state) and no longer expected reward. The authors also found that a revised TD model that included a belief state, which tracks the probability of being in each state produced PE signals that resembled dopamine RPEs. In line with this, others found that administration of L-DOPA to healthy individuals increased model-based over model-free choice (Wunderlich and others 2012). However, other work did not find model-based state PEs in midbrain or striatal dopaminergic regions (Gläscher and others 2010).

It is important to note here that the encoding of RPEs is not limited to the VTA/SN as other regions show responses depending on the nature for reward (Garrison and others 2013; Sescousse and others 2013). Specifically, whereas monetary RPEs were additionally observed in the orbitofrontal cortex, food and erotic rewards additionally engaged the anterior insula and the amygdala (Sescousse and others 2013).

## Dopamine Beyond RPE Coding

### Salience

In addition to its role in RPE coding, there is incomplete evidence that dopamine encodes salience, that is, the extent that a stimulus is particularly noticeable (Schultz 2016b). Focusing attention on stimuli that stand out could be evolutionary advantageous, as it directs attention to those stimuli that are likely to be of importance, for example, noticing a potential predator. It is important to note that RPE coding and salience are not mutually exclusive as the experience of any type of PE, including RPEs, is salient. As such, salience accounts propose a broader role for dopamine than RPE coding. Different types of salience have been defined and we consider these below, taking the view that the different forms of salience may relate to the extent to which a stimulus has been processed. In addition, we discuss the proposed role of dopamine in signaling specific salient events, including identity PEs and novelty.

### Physical Salience

The debate on the role of dopamine in attributing salience focuses in part on whether salience attribution is limited to events that are likely associated with rewards. One line of evidence shows that physically salient sensory stimuli, such as tones and lights, evoke very rapid (50-110 ms), phasic excitations in dopamine neurons (Comoli and others 2003; Dommett and others 2005). This rapid response does not allow detailed identification and evaluation of the stimulus and is therefore unlikely to provide information about a potentially associated reward, although salient and novel stimuli might become erroneously associated with reward (Fiorillo 2013). Novel and physically salient stimuli might, however, be inherently rewarding as they provide unexpected, new information, that might be of value for adaptive behavior (e.g., noticing a brightly colored object in a tree that might indicate an appetitive food; Daw and others 2002b; Reed and others 1996).

### Novelty

As introduced above, a particular type of salient stimuli that recruit dopaminergic responses relate to novelty (Rangel-Gomez and Meeter 2016). For instance, microdialysis studies showed that novel stimuli can evoke dopamine release (Bassareo and others 2002). Dopamine neurons increase their responses in the face of novelty; once novel stimuli become familiar and are not reinforced, dopamine responses habituate (Schultz, 1998). This raises the question whether novelty responses occur purely because of their salient properties. In line with this notion, pharmacological dopaminergic challenge can speed up, and enhance early novelty detection, but does not affect further processing of novel stimuli (see Rangel-Gomez and Meeter 2016, for a review). Bunzeck and Düzel (2006), however, found that whereas the fMRI signal in the SN/VTA responded to novel stimuli, no such effect could be found for other types of salience, including rareness and negative emotional valence, suggesting that dopamine might be particularly responsive to novelty. A later study found that SN/VTA responses to novel stimuli only occurred when novel stimuli were unexpected, bearing close resemblance to findings about reward, which show that responses to reward only occur when unexpected, thus signaling a PE. Although the exact role of dopamine in response to novelty is yet to be determined, it has been suggested, that novelty may motivate exploration which could result in higher rewards (Düzel and others 2010; Kakade and Dayan, 2002; Suri and others, 2001, but also see Lisman and Grace 2005).

### Surprise Salience

Surprise salience, often called surprise, reflects the extent to which a more fully processed stimulus is unexpected (Ungless 2004). As such it operates at a cognitive rather than at a (purely) perceptual level. Surprise can, for instance, denote the magnitude of the PE, independent of its valence (positive/negative). It is thought that this surprise (or *unsigned*) PE signal indicates the degree to which an outcome is unexpected, independent of its sign, and thereby controls the rate of learning (Pearce and Hall 1980), whereas the signed RPE signals the extent to which an outcome is better or worse than expected (Rescorla and Wagner 1972; Sutton and Barto 1998).

A recent meta-analysis across human fMRI studies revealed support for a surprise-encoding network, including the anterior cingulate cortex (ACC), insula and dorsal striatum (Fouragnan and others 2018). Neurophysiological evidence suggests that unsigned PEs, are mainly coded in the cortex, including the dorsal ACC (Hayden and others 2011). This finding is confirmed in human studies that observed prefrontal PE coding in causal learning tasks, in the absence of explicit rewards (Corlett and others 2004; Fletcher and others 2001; Turner and others 2004). Although these brain regions receive dopamine projections (Esber and others 2012) these findings cannot allow inference about the role of dopamine in the encoding of surprise. To identify a potential role of dopamine in coding unsigned PEs (in this case, responses that were indistinguishable for positive and negative PEs), we used dopaminergic perturbations and showed that the dopamine antagonist sulpiride selectively decreased the encoding of unsigned PEs relative to reward reliability in the human superior frontal cortex (Haarsma and others 2019), but not in the striatum or midbrain, which was specific for RPEs (Diederen and others 2017; Haarsma and others 2019).

It should be noted though that, in this study, all PEs occurred in a reward context and it is not clear whether brain responses that do not distinguish negative and positive PEs might be different from responses to PEs that are unrelated to rewarding outcomes. It is conceivable that the potential sensitivity of dopamine to unsigned PEs is still geared toward rewards and would not occur for unrewarded stimuli (Fiorillo 2013).

### Identity and Sensory PEs

A further interesting observation comes from investigations of brain responses to rewards that are matched in (expected) value but differ in reward type/identity (e.g., receiving an equally valued pear instead of the expected apple). Such identity or sensory PEs engender surprise and can as such be considered salient. Using sensory preconditioning (associations between different neutral stimuli) and optogenetics in rodents, recent work showed that the acquisition of information about transitions between non-rewarding events is also driven by PEs and that, dopamine transients were sufficient to support this type of learning (Sharpe and others 2017). These findings were confirmed by recent work in both humans and rats which observed PE signals when the identity of the expected reward was violated (different odors), but the value was kept identical (Howard and Kahnt 2018; Takahashi and others 2017). Interestingly, in the work by Takahashi and others, dopamine responses to changes in value and identity did not occur in different neuronal populations. In contrast, recent work found distinct RPE and identity PE signals in the human midbrain (Boorman and others 2016). However, studies using cyclic voltammetry to monitor dopamine release failed to observe identity PEs (Collins and others 2016; Papageorgiou and others 2016). More work, including studies on fast phasic responses of dopamine neurons is needed to further investigate a potential for dopamine in signaling identity PEs, and to directly contrast work across different techniques and species.

### Motivational Salience

Motivational salience refers to the quality that drives approach behavior for rewarding outcomes and avoidance behavior for aversive outcomes once the physical salience, surprise, and RPEs associated with a stimulus or option have been processed (Robinson and Berridge 2008). Such salience attribution would occur in between the identification of reward, and the generation of action to pursue it (McClure and others 2003). A role for dopamine in aversive salience is, however, heavily contested (Fiorillo 2013).

While neurons in the non-human primate SN increase their firing rate at very short latencies to unexpected stimuli, independent of whether they were rewards or punishment (Matsumoto and Hikosaka 2009), some have reinterpreted this finding as reflecting the physical intensity of stimuli, not their aversiveness (Fiorillo 2013). Others have found that aversive stimuli increase firing in a minority of midbrain neurons in the SN/VTA at longer latencies (Chiodo and others 1980; Mantz and others 1989). To shed more light on these findings, Ungless and others (2004) studied the properties of midbrain neurons that showed aversive responses and found that these midbrain neurons were not dopaminergic. In addition, the authors observed that neurochemically identified dopamine neurons decreased their firing to aversive stimuli. In addition, Fiorillo (2013) observed evidence that supported the existence of opponent neural representations for reward value and aversive outcomes (punishment), which the author concluded to be indicative of the existence of four types of value-sensitive neurons corresponding to reward-ON, reward-OFF, aversive-ON, and aversive-OFF of which only reward-ON was clearly dopamine-mediated (Fiorillo 2013). This is in line with earlier work that showed that motivationally salient events such as the unexpected omission of reward and the unexpected presentation of a stimulus predicting reward omission inhibit dopamine neurons (Tobler and others 2003). Finally, it has been argued that the relieving omission of an expected aversive stimulus can be considered a reward and might therefore evoke dopaminergic responses (Daw and others 2002a; Solomon and Corbit 1978). As such, it appears that dopamine responds selectively to (potentially) positively valenced outcomes, which is formulated in the notion of incentive salience (Robinson and Berridge 2008).

Robinson and Berridge (2008) suggested that mesolimbic dopamine is selectively involved in attributing incentive salience to potential objects or options to guide approach behavior, and that it has no role in RPE coding. Specifically, the authors argue that blocking dopamine selectively inhibits reward-seeking actions, without affecting valuation and the associated RPE of an outcome. This is in strong contrast to the overwhelming evidence for dopamine in RPE coding, and it has been argued by many that dopamine plays a dual role, which guides learning from RPEs and ongoing approach behavior (McClure and others 2003; Schultz 2016b).

Overall, there is relative consensus that mesolimbic dopamine plays a role in the attribution of physical and surprise salience (Schultz 2016b). It is, however, contested whether dopamine processes (motivationally) salient stimuli that are unlikely to be rewarding (i.e., aversive), and whether salience and PE accounts are mutually exclusive (Daw and others 2002b; Robinson and Berridge 2008).

### Integrating RPEs and Salience Accounts of Dopamine

**Figure 6.**
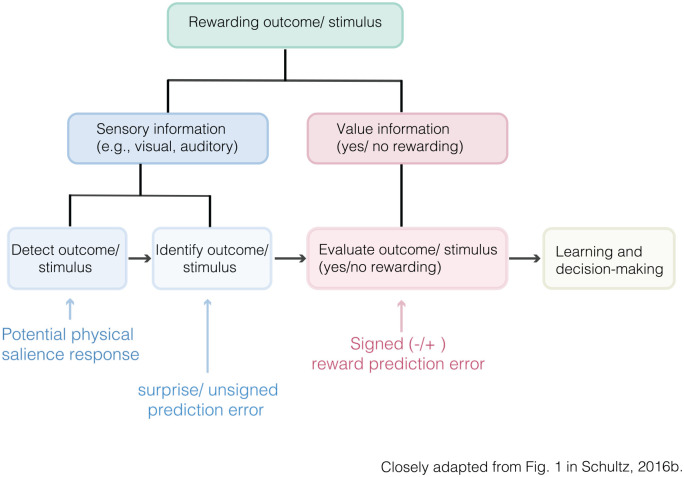
Sequel identification of rewarding outcomes/stimuli.

There are several accounts integrating the proposed roles of dopamine in reward prediction (error coding) and salience. For instance, Schultz (2016b) concluded that dopamine neurons have a “two component response” which integrates accounts of physical salience and RPE coding. Specifically, the first, rapid, component consists of a transient unselective response to a large variety of unexpected stimuli or events, whereas the later, less transient, response signals the occurrence of an RPE (Fig. 6).

In addition, formal learning models such as hybrid RW-PH RL models (Box 1) include a role for surprise as well as the RPE. Here, RPEs drive the trial-wise extent of learning, whereas surprise drives changes in the learning rate across time. Importantly, such hybrid models better predict individuals’ learning behavior than these models alone (Diederen and Schultz 2015; Li and others 2011).

Furthermore, investigations in learning models that include choice behavior (Box 1), indicate that increases in dopamine activation resulting from increases in positive PEs increases the likelihood of choosing an action that leads to reward (Sutton and Barto 1998). Consistent with this, in addition to the phasic response of dopamine neurons in the SN/VTA, dopamine release in the striatum facilitates synaptic plasticity and can directly modulate reward-seeking behavior (Phillips and others 2003; Wickens and others 2003). Thus, it seems that accounts of physical salience, surprise, and incentive (but not aversive) motivational salience, are compatible with RPE accounts as each of these processes appear to be integrated (Daw and others 2002b; Schultz 2016b).

Drawing on recent advances in artificial intelligence, Wang and others (2018) proposed a neurologically plausible meta-reinforcement account where dopamine-driven synaptic plasticity can train a more general and efficient learning system in the prefrontal cortex, allowing it to generalize its learning across different tasks and contexts. The authors carried out a set of simulations that provided support for this account, however, work involving experimental data is required to further test this model.

In addition, some authors have hypothesized that dopamine neurons serve a far more general function in signaling the expectation of (any) information (Bromberg-Martin and Hikosaka, 2009) or signaling errors in any type of prediction where value is only one of the dimensions (Langdon and others 2018; Takahashi and others 2017). The latter bears similarity to the notion that dopamine signals PEs independent of the domain in which these errors occur, thus allowing them to support a broader range of learning.

When combining findings, and theories of dopamine function to date, there appears to be little doubt that dopamine encodes RPEs (but see Friston and others 2015), whereas the hypothesized role in salience coding is slightly more contested. The main question though is whether dopamine *uniquely* codes RPEs, or whether this is one of the (many) functions of dopamine. In light of the work discussed above, the most pressing enquiry is to establish whether dopamine neurons might compute any type of PE, independent of its domain, and how dopamine neurons interact with other brain systems to support other types of learning. The first question could be addressed by testing PEs in different domains, using tasks in which value is held constant or absent. In addition, it would be important to test for several types of PEs at the same time to scrutinize accounts of “multidimensional” PE signaling (Langdon and others 2018).

### Dopamine PEs as a General Mechanism for Learning and Inference

Recent theories relating to the predictive processing framework and active inference have postulated an entirely different role for dopamine (Bastos and others 2012; Friston 2010; Friston and others 2012; Friston and others 2015). According to these theories, which are embedded in Bayesian (inference) models and based on early cybernetic theories, the brain is a predictive “machine” that updates its “model of the world” when PEs occur and its expectations are violated (Ashby 1952; Bayes and others 1763). Note that these accounts differ from Bayesian inference models as the latter characterize “the (computational) problem” that individuals are trying to solve, without making any explicit claims about their neurocognitive architecture (Griffiths and others 2012; Jacobs and Kruschke 2011). In contrast, predictive processing theories often additionally propose specific ways in which Bayesian inference may be implemented in the brain.

Although these ideas resemble RL accounts of learning, these novel theories differ in some major respects. First, they go beyond reward learning, seeing PEs as a generic model for inference and learning (termed belief updating) across sensory and cognitive domains. Furthermore, in these models behavior is optimal, not when reward is maximized, but rather when surprise (or the PE) is minimized. Moreover, they state that dopamine does not code an RPE, but rather codes the precision or reliability of the PE. These models also directly link perception and beliefs with both engaged in making sense of inputs by inferring their causes, an idea that goes back to Von Helmholtz (Von Helmholtz 1867). As PEs indicate unexpectedness in these models, they have clear links with salience accounts of dopamine.

In the predictive processing framework, there is a hierarchy where lower-level PEs signal violations of a sensory nature, while higher order PEs signal violations of beliefs about the probabilistic structure of the environment and its volatility (inverse stability; Friston and others 2014). PEs emitted by a lower-level system becomes the input for a higher level system, whereas feedback from the higher-level system provides the prior beliefs for the lower level system.

The active inference account extends predictive coding into the domain of action and motor control (Friston and others 2012; Friston and others 2015). In simple terms, in this model, surprise can not only be minimized by improving one’s “model of the world” but also by those actions that have predictable outcomes. More formally, perception and action can, respectively, minimize exteroceptive and proprioceptive PEs. This helps individuals to avoid exchanges with the environment that might be harmful (see Friston and others 2012 and Friston and others 2015 for details).

The precision weighting of PEs has been suggested to be dopamine dependent and to ensure that neural systems encoding predictions errors respond more strongly when new information is more reliable (i.e., minimizing surprise) and hence more informative. Lower precision can result from unclear predictions (e.g., early in learning), from noisy perceptual stimuli (e.g., conditions of poor visibility), high variability in the association between different outcomes, and changes in previously learnt associations or environmental volatility (Adams and others 2013; Bastos and others 2012; Friston 2009). Whereas previous work on reward learning has shown that PEs are coded relative to their precision (uncertainty), and that dopaminergic perturbations can modulate the precision-weighting process, a key distinction is that precision weighting is only included in some RL models (e.g. the Pearce-Hall model), whereas it is a key element of Bayesian inference models.

Although many studies have demonstrated support for approximate Bayesian inference, there is little direct experimental evidence to substantiate predictive coding, processing, and active inference frameworks. Some preliminary work using fMRI and PET found that belief updating activated the human VTA/SN and striatum independent of surprise or RPEs, and that these brain responses correlated with midbrain dopamine D2/D3 receptor availability and striatal dopamine release capacity (Nour and others 2018; Schwartenbeck and others 2016). This however contrasts with earlier work that specifically implicated the anterior cingulate cortex in belief-updating (O’Reilly 2013). Other work has more explicitly investigated the proposed hierarchical nature of PE coding, and revealed that whereas precision-weighted low-level PEs (stimulus associations) were coded by the VTA/SN, a higher level precision weighted PEs (expected changes in stimulus associations) engaged brain areas thought to be modulated by the neurotransmitter acetylcholine (Diaconescu and others 2017; Iglesias and others 2013; Payzan-LeNestour and others 2013; Yu and Dayan 2005).

Although these studies provide initial support for a role of dopamine in coding PEs beyond reward, and playing a critical role in the building and updating of internal models of the world, it is important to establish the role of dopamine in this process as the SN/VTA contain dopaminergic and non-dopaminergic neurons (Nair-Roberts and others 2008). Furthermore, the findings that higher level PEs occurred in areas modulated by acetylcholine suggests that the dopamine might not be the primary neurotransmitter for coding PEs across the hierarchy.

### PEs and Other Neuromodulary Systems

It is well known that dopamine interacts with other neurotransmitters, and that there is evidence of other neurotransmitters coding PEs. As a comprehensive overview of this topic is beyond the scope of this review, we illustrate this with the use of a few, select, examples.

Although much of the evidence is indirect, glutamate has frequently been associated with PE coding (see Pennartz and others 2000 and Lapish and others 2006, for theoretical accounts). In line with the notion that NMDA receptors drive dopamine responses to positive PEs, Jocham and others (2014) found that the NMDA antagonist memantine reduced positive but not negative PEs in the human striatum. In contrast, using the NMDA receptor antagonist ketamine, Corlett and others (2006) observed perturbed PE coding in the frontal cortex, but not in the striatum. Using a different approach, White and others (2015) found that glutamate in the SN, measured using MR spectroscopy, correlated with PE signals, in healthy individuals.

Noradrenaline and serotonin have also been implicated in PE signaling. Bouret and Sara (2004) found that noradrenergic neurons of the locus coeruleus in rats showed an RPE response similar to that observed for dopamine. Serotonergic neurons on the other hand, coded the magnitude of the PE (i.e., unsigned PEs) but did not differentiate between positive and negative PEs (Matias and others 2017).

Other neurotransmitters have been shown to interact with dopamine neurons to facilitate learning. For instance, GABA (γ-aminobutyric acid) neurons inhibit dopamine neurons when reward is expected, with contributes to the calculation of RPEs (Eshel and others 2015), a finding that was confirmed by Sharpe and others (2017). Furthermore, Kempadoo and others (2016) showed a tight relationship between dopamine and noradrenergic in facilitating learning. Finally, acetylcholine and norepinephrine have been proposed to signal uncertainty (Yu and Dayan 2005), which is a key component in learning and novel accounts of inference (see previous section).

## Clinical Implications: The Case of Psychosis

A deeper understanding of the precise role of dopamine in PE coding and related functions is not merely of theoretical interest as dopamine dysfunction has been implicated in a range of diseases, including Parkinson’s disease, Huntington’s disease, substance use disorders, depression, anxiety disorder, and attention hyperactivity deficit disorder. There is particularly powerful evidence linking altered dopamine function to psychosis in the context of illnesses such as schizophrenia (Gatt and others 2015; Kollins and Adcock 2014; Nestler and Carlezon 2006). Although it is unclear how exactly altered dopamine can give rise to the symptoms of psychosis, several mechanisms have been put forward. Several proposed mechanisms have drawn on alterations in one or more of the dopaminergic mechanisms discussed in this review. As a broader discussion is out of the scope of this review, here we will provide a brief overview of dysfunctions in RPE coding and related concepts in psychosis, and some of the theories that have been put forward to link these dysfunctions to psychosis.

Psychosis has consistently been linked to increased presynaptic dopamine in the striatum (Howes and others 2012), with the dopaminergic alteration possibly preceding the onset of clinical-level psychosis (Howes and others 2011). In addition to these findings, people with psychosis present with an increased density in striatal dopamine D2 receptors, and alterations in genes involved in dopamine function (for a recent review, see McCutcheon and others 2019). Given this, and the fact that the primary treatment for psychosis is dopamine blockade, there has been a growing interest, beginning perhaps with the work of Robert Miller (1976) in embedding the understanding of the basic neuroscience of dopamine into models of psychosis. Indeed, multiple theorist have referred to dopamine as the final common pathway to psychosis (Howes and Murray 2014; Maia and Frank 2017), an account that was supported in a recent review (Valton and others 2017).

Psychosis has been associated with dysfunctions in a number of the dopamine-mediated processes described above (see Deserno and others 2013; Radua and others 2015; Maia and Frank 2017, for overviews). In brief, people with psychosis show attenuated behavioral and neural responses to reward predicting cues, whereas responses to neutral (or irrelevant) cues are increased, suggesting that these individuals experience difficulty identifying predictors of valuable outcomes. In the domain of RL, both patients on and off dopaminergic antipsychotic medication experience difficulties learning from positive RPEs, which is paralleled by attenuated coding of neural RPE signals, while learning from negative RPEs and their accompanying neural responses are preserved.

An influential attempt to explain how dopaminergic alterations may produce psychosis uses the notion of aberrant salience (Kapur 2003). Simply put, the idea is that an erratic phasic dopaminergic signal is experienced as an altered experience of the significance of environmental events and stimuli, which in turn drives a new appraisal of one’s environment and the ensuing altered, and, apparently irrational and inexplicable beliefs (delusions). This “aberrant salience” framework has inspired subsequent cognitive and neuroimaging work supporting the view that irrelevant stimuli may be imbued erroneously with salience in people with psychosis (Murray and others 2008; Roiser and others 2009). However, there is much that remains unexplained by this initial theory and later models have sought to develop more comprehensive explanations of how dopamine function may drive shifted experiences of the world. In this regard, the important insights offered by studies of its role in PE-driven RL have proven very fruitful.

Recent mechanistic accounts have extended previous theories to explain how altered dopamine may give rise to both the positive (hallucinations and delusions) and negative symptoms of psychosis (e.g., affective flattening, alogia, and avolition; Deserno and others 2013; Maia and Frank, 2017). In brief, the authors proposed that reduced dopamine firing for relevant stimuli underlies negative symptoms, whereas an increase in spontaneous phasic dopamine release leads to excessive responses to neutral stimuli and PEs. Deserno and others (2013) furthermore hypothesized that aberrant PEs encode non-salient events as surprising, which drives aberrant learning resulting in these events being imbued erroneously with high incentive values, which can lead to positive psychotic symptoms. Negative symptoms on the other hand, are thought to result from a failure to use PEs to obtain accurate estimates of value. For a detailed mechanistic account at the computational and neurobiological level, see Maia and Frank (2017).

In recent years, it has been increasingly theorized that altered Bayesian inference could explain the symptoms of psychosis (see Valton and others 2017 and Heinz and others 2019, for an overview). Some of these accounts are (relatively) agnostic about the neural mechanisms underlying the hypothesized deficits in inference (Fletcher and Frith 2009; Valton and others 2017). Others have specified how altered Bayesian inference might be implemented in the brain by incorporating principles of predictive coding, predictive processing or active inference (Adams and others 2013; Jardri and Deneve, 2013). In multiple of these accounts, the idea is that the critical impact of dopaminergic perturbation lies in a change in the experienced precision of the PE signal, giving it undue weight and making it possible to change even long held and widely shared beliefs into the odd beliefs observed in psychosis. To date, support for these models has mainly been provided through simulations, stressing the need for more experimental work in individuals with psychosis (see Valton and others 2017 and Heinz and others 2019, for a summary of preliminary evidence).

It is important to remember that these “bayesian predictive coding” accounts entail the conjoining of two separate theoretical approaches. One (Bayesian inference) relates to a system’s computational goal while the other, predictive coding, is an “algorithmic motif” (Aitchison and Lengyel 2017). A predictive coding system does not necessarily engage in Bayesian inference while Bayesian inference does not necessarily entail predictive coding (see Aitchison and Lengyel 2017 for a comprehensive discussion). Thus, many of the assumptions of the above approaches have yet to be empirically validated. Nonetheless, they do offer opportunities to credibly link emerging insights into dopaminergic contributions to PE to our attempts to understand the complex and baffling symptoms and subjective experiences of psychosis.

## Conclusions

A wealth of studies has confirmed the role of dopamine in RPE coding, using a large range of different techniques and validation across different species, including rodents, monkeys, and non-human primates. It is, however, likely that dopamine has an additional role in signaling the amount of surprised associated with a rewarding outcome or stimulus, which can well be integrated with the RPE framework. Finally, novel models suggest an entirely different role for dopamine in PE coding across the whole brain, which might be exceptionally important for understanding clinical conditions associated with altered dopamine processing such as psychosis. It will be crucial to investigate these novel accounts in future studies using experimental designs and data, and to further the work on the link between these models and clinical conditions.
